# Implications of Spatiotemporal Regulation of *Shigella flexneri* Type Three Secretion Activity on Effector Functions: Think Globally, Act Locally

**DOI:** 10.3389/fcimb.2016.00028

**Published:** 2016-03-09

**Authors:** F.-X. Campbell-Valois, Stéphanie M. Pontier

**Affiliations:** ^1^Department of Chemistry and Biomolecular Sciences, University of OttawaOttawa, ON, Canada; ^2^Independent ResearcherOttawa, ON, Canada

**Keywords:** *Shigella*, type three secretion apparatus, type three secretion system, effectors, enteropathogens, host-pathogen interactions, signal transduction

## Abstract

*Shigella* spp. are Gram-negative bacterial pathogens that infect human colonic epithelia and cause bacterial dysentery. These bacteria express multiple copies of a syringe-like protein complex, the Type Three Secretion apparatus (T3SA), which is instrumental in the etiology of the disease. The T3SA triggers the plasma membrane (PM) engulfment of the bacteria by host cells during the initial entry process. It then enables bacteria to escape the resulting phagocytic-like vacuole. Freed bacteria form actin comets to move in the cytoplasm, which provokes bacterial collision with the inner leaflet of the PM. This phenomenon culminates in T3SA-dependent secondary uptake and vacuolar rupture in neighboring cells in a process akin to what is observed during entry and named cell-to-cell spread. The activity of the T3SA of *Shigella flexneri* was recently demonstrated to display an on/off regulation during the infection. While the T3SA is active when bacteria are in contact with PM-derived compartments, it switches to an inactive state when bacteria are released within the cytosol. These observations indicate that effector proteins transiting through the T3SA are therefore translocated in a highly time and space constrained fashion, likely impacting on their cellular distribution. Herein, we present what is currently known about the composition, the assembly and the regulation of the T3SA activity and discuss the consequences of the on/off regulation of T3SA on *Shigella* effector properties and functions during the infection. Specific examples that will be developed include the role of effectors IcsB and VirA in the escape from LC3/ATG8-positive vacuoles formed during cell-to-cell spread and of IpaJ protease activity against N-miristoylated proteins. The conservation of a similar regulation of T3SA activity in other pathogens such as *Salmonella* or Enteropathogenic *Escherichia coli* will also be briefly discussed.

## Introduction

*Shigella* spp. (e.g., *S. flexneri, S. sonnei, S. dyssenteriae*, and *S. boydii*) are gram negative enteropathogen bacteria that are closely related to commensal *Escherichia coli*. As such, they are often considered to be *E. coli*. pathovars. *Homo sapiens* are the only known natural hosts of *Shigella* spp. By invading the colonic mucosa, *Shigella* spp. cause dysentery that is characterized by bloody and mucous rich diarrhea accompanied by abdominal cramps. There are about 200 million infection cases annually and ~1.1 million deaths, among which the majority are children under 5 years (Kotloff et al., 1999). Associated to poor sanitation and water quality control (Kotloff et al., 1999; Phalipon et al., 2008; Johansson et al., 2009), the prevalence of the disease is highly correlated with economic wealth. In addition, the etiology of the disease differs between low- and high-income countries, where *S. flexneri* and *S. sonnei* prevail, respectively. Potential reasons for this remarkable phenomenon are discussed in detail elsewhere (Thompson et al., 2015).

*Shigella* spp. pathogenicity essentially depends on a large virulence plasmid of ~200 kb that is also found in enteroinvasive *E. coli* (EIEC). This virulence plasmid (Buchrieser et al., 2000; Venkatesan et al., 2001; Zhang et al., 2003; Jiang et al., 2005), and the chromosomes (Lukjancenko et al., 2010; Onodera et al., 2012) of many *Shigella* spp. have now been sequenced. Still, the majority of what we know concerning the infectious cycle of *Shigella* spp. and the molecular determinants of their pathogenicity comes from studies on *S. flexneri*, namely strains M90T (serotype 5a), 2457T (serotype 2a), and YSH6000 (serotype 2a) either in *in vitro* culture of immortalized intestinal cells, or from the infection of various animal hosts, including primate, rabbit, guinea pig, or mouse (Sansonetti et al., 1983; Sansonetti and Arondel, 1989; Martino et al., 2005; Shim et al., 2007; Arena et al., 2015). While none of these experimental systems constitute a natural *Shigella* host, they have nevertheless provided many insights about the inflammatory response component of shigellosis. This is particularly true of the rabbit ileal loop model (Sansonetti et al., 1983; Schnupf and Sansonetti, 2012; Puhar et al., 2013).

The infectious cycle of *Shigella* spp. consists in several consecutive steps. Upon their adhesion to host cells, *Shigella* spp. use genes expressed from their virulence plasmid to trigger their uptake by otherwise non-phagocytic epithelial cells, access their host cell cytoplasm and then, eventually spread to neighboring cells (reviewed in Valencia-Gallardo et al., 2015). The virulence plasmid also allows the bacteria to survive inside and kill macrophages (Zychlinsky et al., 1992; Fernandez-Prada et al., 2000; Suzuki et al., 2014), and perturb the function of T and B cells (Konradt et al., 2011; Salgado-Pabón et al., 2013; Nothelfer et al., 2014). Protein products of many genes harbored on the virulence plasmid are necessary for the assembly of a nanomolecular machine named the Type Three Secretion Apparatus (T3SA) (Burkinshaw and Strynadka, 2014). Also known as injectisome, this T3SA plays an essential role in most of *Shigella* invasion steps. The T3SA spans the bacterial inner and outer membranes adopting roughly the shape and function of a syringe. T3SA have a narrow conduit in their center that permits the secretion of proteins. In the initial stage of T3SA activation that takes place after initial contacts with the PM, a first class of protein called translocators are secreted. The translocators assemble to form a pore also called translocon across the host membrane. A second group of proteins called effectors then transit through the T3SA and ultimately through the pore to be delivered in the host cytoplasm. Simultaneously the host PM engulf the bacteria through a process requiring actin microfilaments remodeling, similarly to what is seen is regular phagocytosis (reviewed in Ménard et al., 1996; Carayol and Tran Van Nhieu, 2013; Valencia-Gallardo et al., 2015). The bacterial uptake is completed when bacteria are found in closed vacuoles. The T3SA is also necessary for subsequent rupture of these vacuoles (Blocker et al., 1999; Page et al., 1999; Schuch et al., 1999; Paz et al., 2010). Once in the cytoplasm *Shigella* spp. use the outer membrane protein IcsA (also known as VirG) to form actin comet tails that enable cytoplasm movement and ultimately, cell-to-cell spreading (Bernardini et al., 1989). The collision of a motile bacterium with the inner leaflet of the PM leads to the formation of a protrusion, which is a double membrane finger-like projection of the PM of the initially infected cells into a neighboring cell. Protrusions resolved into secondary vacuole (Campbell-Valois et al., 2014b; Dragoi and Agaisse, 2014; Kuehl et al., 2015); secretion of translocators and effectors are known to be essential, as well, for the lysis of secondary vacuoles through a process hypothesized to be essentially similar to entry (Page et al., 1999; Schuch et al., 1999). The ensuing release of bacteria into the cytoplasm of secondary infected cells effectively completes cell-to-cell spreading events.

As yet, the translocators and effectors arsenal of *S. flexneri* is encoded by 32–38 genes (Buchrieser et al., 2000; Ogawa et al., 2008; Parsot, 2009). While the N-terminal region of most of these effectors appears required for their targeting to the T3SA, their level of homology do not allow the identification of any clear consensus targeting sequence (Ramamurthi and Schneewind, 2003; Ghosh, 2004; Lilic et al., 2006). In addition, the structural stability of several of these effectors and their efficient targeting to the T3SA can be dependent on the formation of a complex with their cognate chaperone protein (reviewed in detail elsewhere Burkinshaw and Strynadka, 2014). The nine effectors that binds the chaperone Spa15 were recently shown to harbor a conserved chaperon binding domain required for efficient secretion and conserved across many pathogen species (Costa et al., 2012). However, most of the effectors, whose expression is up regulated when T3SA are active, do not seem to necessitate any chaperone (Parsot, 2009).

In this review, we first focus on the current knowledge concerning the assembly and the structure of the T3SA. We then describe the evidences indicating that *Shigella* T3SA activity oscillates depending on the adhesion of bacteria to the host PM. In the third part, we discuss the consequences of this dynamic activity of the T3SA on the properties and functions of *Shigella* effectors. Finally, we relay this model to recent data concerning effector functions and discuss its extension to other T3SA-bearing pathogens.

## Expression, assembly, and structure of *Shigella* T3SA

**Figure 1:**
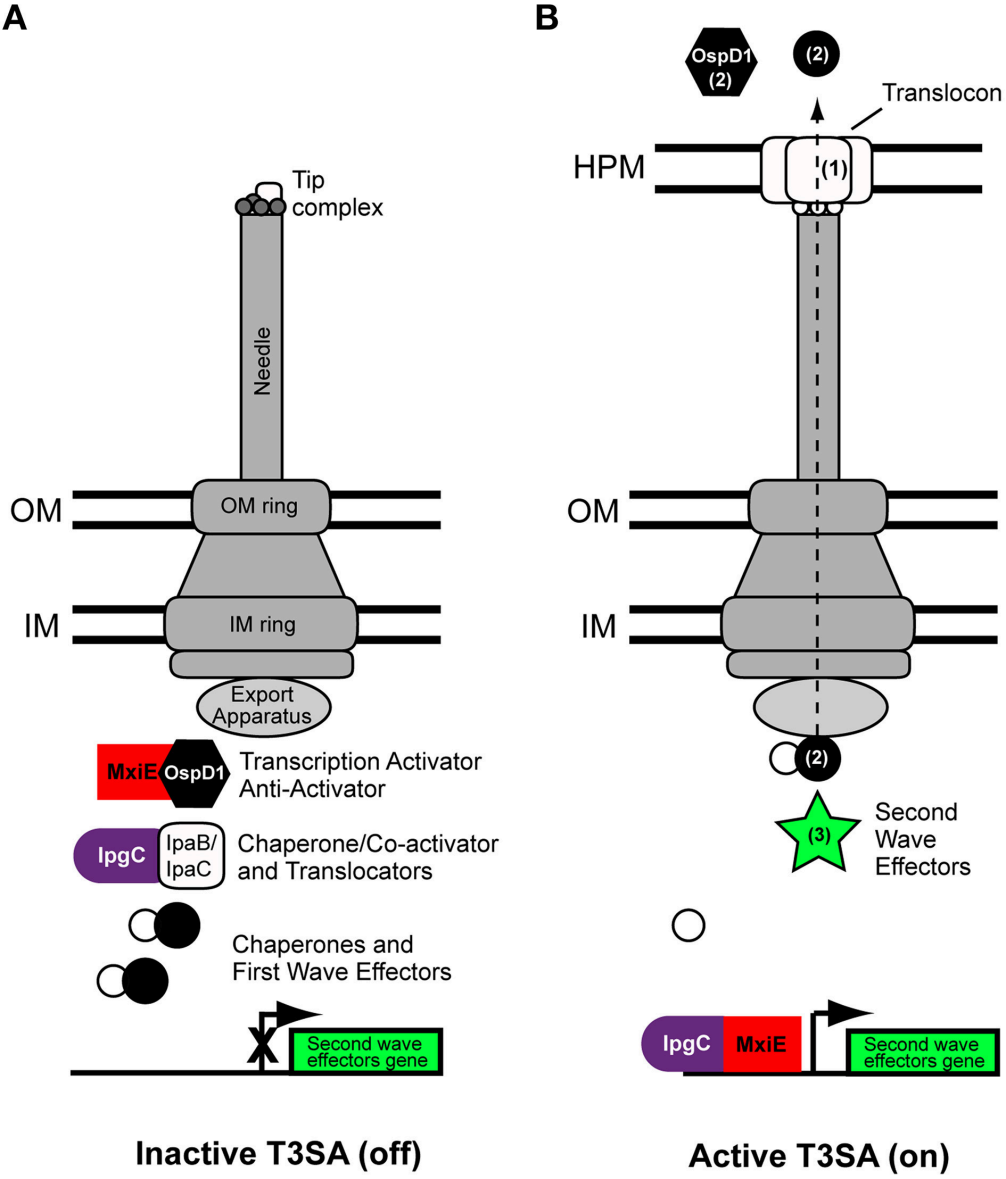
**Scheme of the type three secretion apparatus of *Shigella flexneri***. Basic scheme of *Shigella flexneri* T3SA at permissive temperatures (e.g., 37°C) in the inactive **(A)** and active states **(B)**. The tip complex is composed of IpaD (grey circles) and IpaB (white rectangles) adopting a closed conformation and an open conformation in the inactive and active states, respectively. Activation of secretion leads to MxiE-IpgC-dependent expression of second wave effectors. The dashed arrow indicates the route followed by translocators and effectors during secretion. They travel through a conduit located at the center of the T3SA that comprises successively the sorting platform, the inner membrane ring, rod protein (not visible on this scheme), the outer membrane ring, the needle, and translocon. Numbers in parenthesis in the right panel indicate the secretion order of translocators, first wave and second wave effectors. Labelings of bacterial cytoplasmic complex components are indicated from left to right. IM, inner membrane; OM, outer membrane; HPM, host plasma membrane.

The expression of T3SA is controlled at the transcriptional level. Essential genes for assembly of T3SA are located in two juxtaposed, but inversely oriented operons, located in the center of the virulence plasmid: the *mxi/spa* operon (approximately 20 kb length and 26 genes) and the *ipaABCD* operon (~10 kb length and 10 genes) (Buchrieser et al., 2000). Importantly, the transcription of the T3SA is tightly associated and synchronized to those of the effector proteins. Indeed, at temperature above >32°C, inhibition by the nucleoid factor H-NS is relieved (Maurelli and Sansonetti, 1988; Falconi et al., 1998, 2001), triggering a signaling cascade implicating transcription activators VirF and VirB that induces the expression of *mxi/spa* and *ipaABCD* operons (Tobe et al., 1991; Kane and Dorman, 2012). The output of this cascade consists in the formation of an intracellular store of translocators and so-called first wave effectors with their cognate chaperones (Ménard et al., 1994b), and the assembly of T3SA (Figure 1). Therefore, bacteria at permissive temperatures display at their surface inactive T3SA that can be switched to the active state upon contact with host cells, allowing almost instantaneous secretion of prestored translocators and effectors (Enninga et al., 2005).

The inactive T3SA is hierarchically assembled in the bacterial membranes (reviewed in detail elsewhere Burkinshaw and Strynadka, 2014) (Figure 1). Proteins MxiG/MxiJ and MxiD/MxiM, constituting the inner and outer membrane rings of the basal body of the T3SA, respectively, are assembled first (Hodgkinson et al., 2009). MxiA, Spa13, and Spa47 and the sorting platform, which is composed of Spa33, MxiK, and MxiN (Morita-Ishihara et al., 2006; Lara-Tejero et al., 2011; Hu et al., 2015), associate with the cytoplasmic face of the inner membrane ring where they can recognize proteins targeted to the T3SA. Remarkable high resolution electron microscopy images of the T3SA of *Shigella*, recently provided compelling evidence about the composition and function of the cytoplasmic components of the T3SA, including the sorting platform (Hu et al., 2015). On the basis of its *Salmonella* homolog PrgJ, the rod protein MxiI is hypothesized to associate with the socket in the upper part of the inner membrane ring and contribute to regulating secretion of MxiH (Marlovits et al., 2006), which homopolymerizes to form the needle of the syringe (Demers et al., 2013). Interaction of Spa32 with Spa40 is also essential for the formation of needles (Botteaux et al., 2008, 2010). Spa32, the homolog of YscP in *Yersinia pestis* (Journet et al., 2003), acts as a molecular ruler and is secreted when the needle reaches the correct length (Botteaux et al., 2008). A small fraction of the total cellular pool of IpaB and IpaD is then secreted, but remains associated with the needle, hence forming the tip complex. In the absence of activation signal, the tip complex is closed and composed of one molecule of IpaB and four molecules of IpaD (Veenendaal et al., 2007; Epler et al., 2012; Cheung et al., 2015). The association of the closed conformation of the tip complex with the needle is a hallmark of inactive T3SA. In contrast, T3SA devoid of this normal tip complex, which are obtained by deletion of the *ipaB* or *ipaD* locus, are constitutively active (Ménard et al., 1993, 1994a). In the case of the *ipaB* mutated strain, the open conformation of the tip complex appeared to be formed of five IpaD molecules (Cheung et al., 2015), but it is likely that total absence of a tip complex would also lead to deregulated secretion. In the inactive state of T3SA, the gatekeeper protein MxiC blocks effectors secretion (Botteaux et al., 2009; Martinez-Argudo and Blocker, 2010). It does so by associating with the entrance of the inner conduit of the T3SA, probably through binding with MxiI (Cherradi et al., 2013). MxiC remains at this position until it is itself secreted, an event that is probably induced by the depletion of the intracellular store of translocators occurring in the active state, and that may involve needle conformational changes (Martinez-Argudo and Blocker, 2010).

## Regulation of the activity of *Shigella* T3SA inside infected cells

The T3SA is activated upon contacting the host cell, likely upon binding of the tip complex to cholesterol and/or sphingolipid molecules composing the host PM (Lafont et al., 2002; van der Goot et al., 2004; Veenendaal et al., 2007; Epler et al., 2009). This activation triggers the secretion of the cytoplasmic fraction of translocators IpaB and IpaC. IpaB and IpaC insert into the host PM to form a pore, or translocon, through which effectors will be transferred into the host cytoplasm (Figure 1) (Edgren et al., 2012). Thus, a first hallmark of an active T3SA state is the adoption of an open conformation by the tip complex or, alternatively, its absence from the needle of active T3SA. Both situations result in the unsealing of the syringe. A second hallmark is the cooperation between the T3SA and the translocon. Importantly, this cooperation is necessary to infect cells, but dispensable for constitutive *in vitro* activity, as for example in the case of *ipaB* and *ipaD* mutant strains.

Upon persistent activation of the T3SA, bacteria intracellular stores of translocators IpaB and IpaC and of the anti-activator OspD1 become depleted (Figure 1). This putatively allows the formation of a complex between the translocator chaperone IpgC and the transcription activator MxiE (Pilonieta and Munson, 2008), which induce the expression of genes harboring a MxiE-box (Mavris et al., 2002a,b; Le Gall et al., 2007; Bongrand et al., 2012). Genes possessing a MxiE-box hence constitute a second wave of effectors that are secreted through the T3SA (Parsot, 2009) (Figure 1).

Transcriptional fusions of MxiE-box containing promoters with β-galactosidase (LacZ) were constructed and used to monitor the T3SA activity of bacteria recovered from infected HeLa epithelial cells (Demers et al., 1998). HeLa cells are not very permissive for cell-to-cell spreading (Tran Van Nhieu et al., 2003), but allow the study of events taking place during the initial uptake and vacuolar rupture. In the absence of secretion activity, such as when bacteria are grown in broth at 37°C, the β-galactosidase activity of MxiE-promoters was nil. In contrast, when bacteria were put in contact with Hela cells, the β-galactosidase activity of MxiE-promoters was induced. The β-galactosidase activity of *Shigella* recovered from HeLa cells was higher at 60 than at 150 min post-entry. The activity at 150 min had in fact decreased to the background level observed in bacteria cultivated in absence of host cells. These results provided the first indication that following entry into epithelial cells, T3SA were inactivated (Demers et al., 1998).

**Figure 2:**
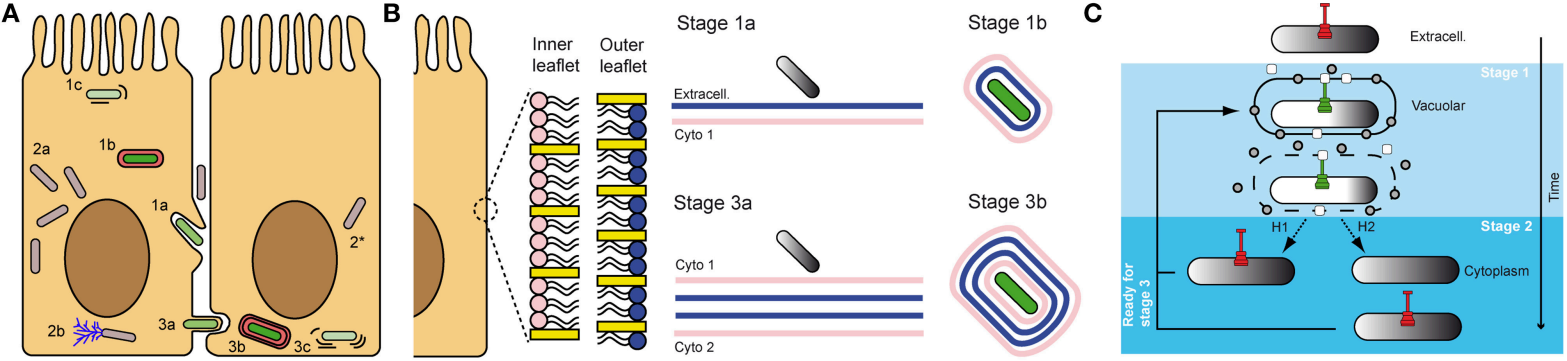
***Shigella* infectious cycle: interaction of T3SA with membrane compartments is key for the regulation of its activity**. The “invade and evade” infectious strategy of *S. flexneri* can be broken down in two phases: **(1)** entry, characterized by residence of bacteria in vacuoles derived from the PM **(1a,b)**, which are ultimately ruptured **(1c)**; **(2)** cytoplasmic residence, where most replication events occur **(2a)** and motility through actin comet formation is possible **(2b)**. After bacteria have reached the cytoplasm, they are in position to iterate this cycle and progressively invade neighboring cells, before evading once again the secondary vacuole. This process is characterized by the formation of protrusions **(3a)** and vacuoles **(3b)** composed of a double membrane derived from the PM in which bacteria reside until their lysis **(3c)**, and escape in the cytoplasm (2^*^). It was demonstrated that secreting bacteria (green) were systematically associated with entry and cell-to-cell spread vacuoles and protrusions derived from the PM, while cytoplasmic bacteria were not actively secreting (gray) **(A)**. Magnification of the inner and outer leaflet of the PM. Density of cholesterol (yellow rectangles) and overall phospholipids composition (pink vs. blue) is variable in both leaflets. Therefore, bacteria are not facing the same biochemical cues when they are performing entry vs. cell-to-cell spread. As mentioned in panel **(A)**, bacteria also face four membranes during cell-to-cell spread instead of two during entry **(B)**. Proposed mechanisms of inactivation of T3SA in intracellular *Shigella*. Grey circles and white rectangles represent secreted tip complex proteins, which are incapable of blocking T3SA conduit. H1 and H2 represent alternative hypotheses for inactivation of T3SA in the cytoplasm of host cells, as described in the text. H1: replenishment of functional tip complex; H2: disassembly of T3SA before vacuole escape and replenishment with inactive T3SA in the host cytoplasm **(C)**.

Use of the green fluorescent protein (GFP) allowed the design of fluorescent Transcription-based Secretion Activity Reporter (TSAR) relying on the MxiE-promoter of *ipaH7.8.* The TSAR allowed for monitoring the T3SA activity inside infected cells in close to real-time fashion (Campbell-Valois et al., 2014a,b). It confirmed the results obtained with the previous β-galactosidase transcriptional fusion (Demers et al., 1998). Indeed, the secretion proved inactivated in the host cell cytoplasm 30–60 min post-entry. In addition, because these experiences were performed in colonic epithelial cell line TC7 (a clone of Caco-2), the dynamic of the TS3A activity during the spreading of bacteria to neighboring cells could be observed. Interestingly, a significant fraction of bacteria that had escaped the entry vacuole were observed to reactivate their secretion between 60 and 120 min post-entry. Based on several lines of evidence, this phenotype was attributed to the fraction of motile cytoplasmic bacteria that had formed protrusions (Figure 2A). For example, non-motile *Shigella* obtained by genetic manipulation (e.g., *icsA* mutant) or treatment with the actin polymerization inhibitor cytochalasin D, both resulted in background level of T3SA secretion activity at 240 min post-entry. In contrast, using a conditional mutant *ipaC* allele that remained trapped in protrusions or in vacuoles that derived from it, or using the F-actin depolymerizing inhibitor jasplakinolide, which induced host cell retraction that causes random collisions between intracellular bacteria and the PM, we demonstrated that interactions of cytoplasmic bacteria with the PM compartments formed during cell-to-cell spread was critical for reactivation of T3SA. These results demonstrate that interactions of cytoplasmic bacteria with the PM formed during cell-to-cell spread were critical for reactivation of T3SA. Fluorescence Recovery After Photobleaching (FRAP) of the TSAR indicated that the secretion activity was induced when bacteria were trapped in protrusions and in a lesser measure in vacuoles, but not in cytoplasmic bacteria (Campbell-Valois et al., 2014b). This study indicated that intracellular *Shigella* undergoes cyclical all-or-none activation of its T3SA depending on interactions with the PM during entry or cell-to-cell spreading steps of the infection cycle. In addition, these results also indicate that endomembrane compartments are likely unable to induce T3SA activation. Whether this phenomenon stems from the biochemical composition of the endomembrane compartments itself, which would fail to activate T3SA due to weaker mutual interactions, or from the infrequent docking of *Shigella* on endomembrane compartments is an open question. It is also possible that T3SA display low level activity or too transient activation in the cytoplasm to be detectable with the TSAR system. Another important question to tackle is the regulatory mechanism of T3SA activity in infected cells and tissue. How T3SA can be activated both during entry and cell-to-cell spread while the bacteria is alternately facing the external face of a single PM and the internal face of a double PM (Figure 2B)? Additionally, what are the mechanisms of inactivation of T3SA in the cytoplasm (Figure 2C)? Concerning the latter question, the most plausible mechanism is the reconstitution of tip complexes composed of newly synthesized IpaB and IpaD capable of plugging T3SA shortly after loss of contacts with the vacuolar membrane. The alternative hypothesis of a partial or complete disassembly of T3SA following bacterial release in the cytoplasm appears less likely, but cannot be completely ruled out yet (Campbell-Valois et al., 2014b).

## A model integrating the influence of the oscillating T3SA activity on the properties and functions of effectors

As delineated above, *Shigella* infectious cycle can be summed up as an “invade and evade” strategy, where bacteria first “invade” cells by triggering their own uptake in epithelial cells (or by not blocking their uptake by professional phagocytes) and “evade” the vacuole formed around them during phagocytosis using membrane-disrupting translocators and effectors. Once bacteria have evaded their vacuole, they are in position to iterate this cycle and progressively invade neighboring cells, before evading once again the secondary vacuole. The observation that the T3SA oscillates between its active and inactive states between two “invade-evade” cycles (Campbell-Valois et al., 2014b) has likely important consequences on the subcellular distribution of bacterial effectors during infection. This subcellular distribution is influenced by three main parameters: the location of secretion, the regulation of secretion, and the diffusion capacity of the effector within the host cytoplasm, either passively through Brownian movement or actively by binding specific host factors or organelles. The cellular cytoplasm is characterized by a high concentration of biomolecules or macromolecular crowding, which considerably impedes the excluded volume of solvent accessible to diffusing proteins, hence decreasing their diffusion rate (Zhou et al., 2008). The macromolecular crowding is heterogeneous and peaks at the vicinity of the host cell PM (Kühn et al., 2011). In consequence, the protein diffusion rate in this region is decreased (Kühn et al., 2011). The formation of protein complexes and the level of cytoskeleton polymerization participate to the heterogeneity of the macromolecular crowding. Therefore, any perturbation in the density of the cytoskeleton network can potentially further restricts protein diffusion. Interestingly, many pathogens such as *Salmonella, E. coli*, and *Shigella*, increase the density of the actin meshwork in their vicinity using T3SA effectors. Specifically, *Shigella* entry and cell-to-cell spreading is characterized by the formation of actin foci or actin rich structures around actively secreting bacteria (Carayol and Tran Van Nhieu, 2013). Specific *Shigella* effectors involved in that process will be discussed later.

**Figure 3:**
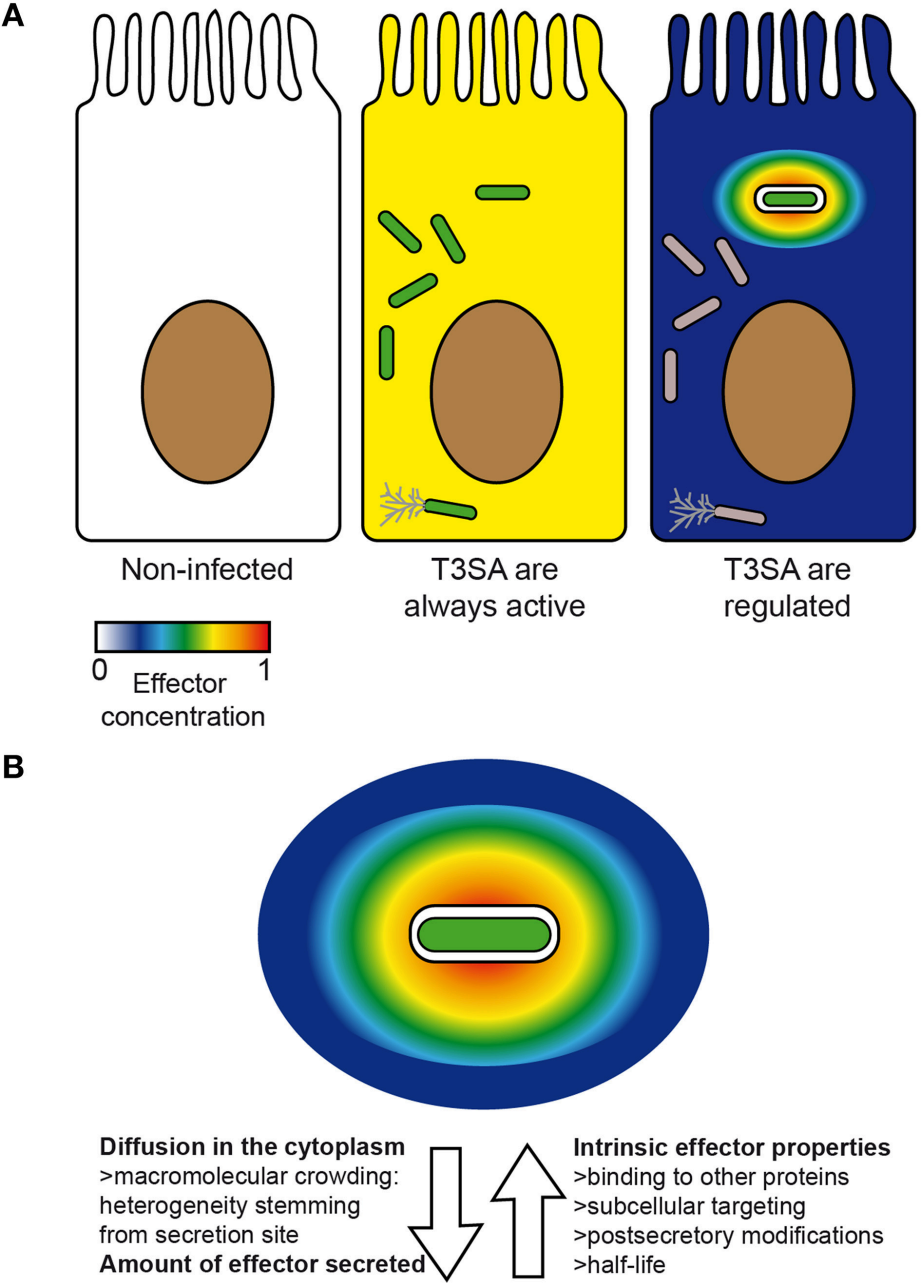
**Factors impacting on the concentration and distribution of effectors inside host cells**. Theoretical concentration and distribution of bacterial effectors in an uninfected cell (left) vs. in infected cells in the case scenario where all intracellular bacteria (center) or only those in vacuoles are secreting (right) **(A)**. An actively secreting bacterium located in a vacuole is represented with its theoretical gradient of effectors. Arrows oriented away and toward the bacteria represent respectively factors favoring or disfavoring rapid and homogenous diffusion of effectors inside host cells **(B)**. Secreting bacteria, green; non-secreting bacteria, gray. Color scale represents concentration of effectors from 0 (min) to 1 (max) (arbitrary unit).

Hence, if the intrinsic properties of effectors as well as their capacity to interact with host protein targets obviously play a determinant role in their function, the site of their secretion is also crucial (Galán, 2009). Furthermore, the realization that the T3SA activity is maximal in PM-derived compartments such as protrusions and bacteria-containing vacuoles strongly suggests that the effective concentration of effectors upon their secretion should follow a very steep gradient (Figure 3A). This prediction was experimentally corroborated by the apparent retention of translocators and effectors in the vicinity of actively secreting bacteria (Campbell-Valois et al., 2014b, 2015). This high effective concentration of effectors should hence potentiate their binding and enzymatic properties, as long as their host protein targets are as well localized in this region.

**Figure 4:**
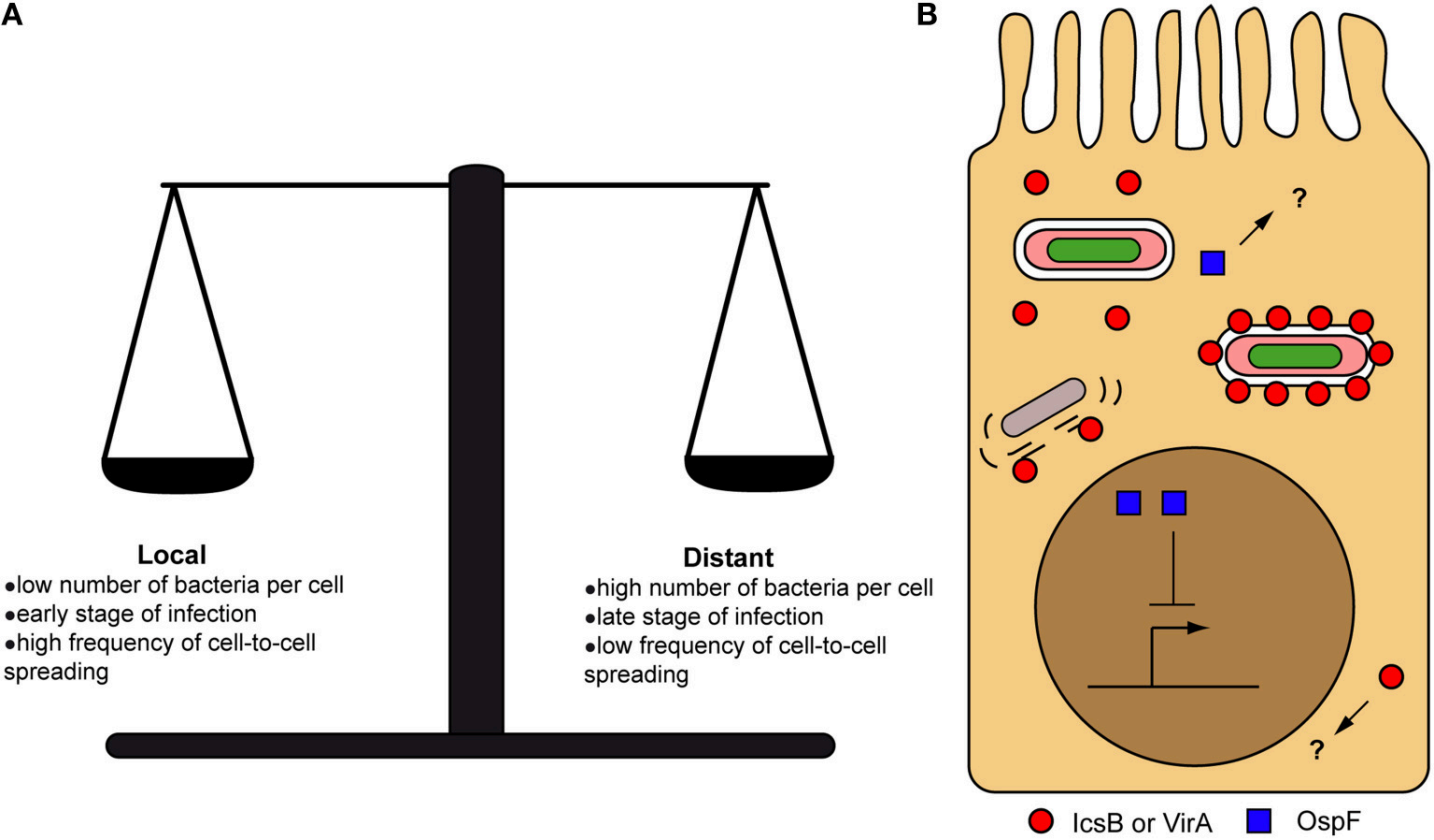
**Local vs. distant action of secreted effectors**. Factors influencing the balance between local and distant action of effectors from their secretion site inside host cells **(A)**. Examples of local and distant action of effectors inside host cells. OspF is acting in the nucleus (distant action) to block the inflammatory response, while IcsB and VirA were recently proposed to act directly on the cell-to-cell spread vacuole (local action) to favor bacterial escape in the cytoplasm. Question marks indicate the possibility that OspF and IcsB/VirA could also have local and distant functions, respectively **(B)**.

Despite the importance of the initial local concentration of effectors, experimental observations of their subcellular distribution have demonstrated that many effectors can nevertheless eventually diffuse from their secretion site, when given enough time (Campbell-Valois et al., 2014b, 2015). Again, the diffusion rate and the final subcellular distribution of these effectors will depend on the properties of the cytoplasm of the (infected) cell and the intrinsic properties of effectors (Figure 3B). Therefore, the local delivery and ensuing diffusion of effectors may confer them two sets of functions: one in the vicinity of secreting bacteria (i.e., local functions) and one upon diffusion across the infected cell (i.e., distant functions) (Figure 4). The balance between local vs. distant functions for a given effector would also be modulated by the actual amount of secreted effector, the relationship between its binding affinity and/or catalytic activity toward its host targets and its stability or half-life. Finally, the amount of actively secreting bacteria within a given infected host cell might also impact on the concentration and therefore the distribution of effectors across infected cells. Distant functions might be favored, when the amount of bacteria and secreted effectors cell rise and/or when the amount of actively secreting bacteria decreases in infected host cells (Campbell-Valois et al., 2014b). In conclusion, this local/distant model of effectors function would provide a passive and nevertheless elegant manner for bacteria to adapt their activity in regard of the bacterial load within host cells.

## Recent progresses in the role of *Shigella* effectors during infection: evidence for and against our model

### Examples of effector acting locally, near their secretion site

Few studies performed on *S. flexneri* support the existence of local function of effectors. A classical example is the local function of IpaB (in collaboration with IpaC) through its translocon-forming ability to induce bacterial uptake and vacuole rupture (Blocker et al., 1999; Page et al., 1999; Schuch et al., 1999; Carayol and Tran Van Nhieu, 2013), which can be contrasted with IpaB distant function in inducing pyroptosis of macrophages once liberated in their cytoplasm (Zychlinsky et al., 1992, 1994). Other effectors implicated in F-actin manipulation, such as IpaA, IpgB1, and IpgB2 are also known to act near or around the actin foci to enable bacterial uptake (Carayol and Tran Van Nhieu, 2013). Phosphatidylinositol-5-phosphate produced by the inositol phosphate phosphatase IpgD is enriched around entry sites (actin foci), suggesting IpgD is acting in the vicinity of its secretion site (Pendaries et al., 2006). However, the reduction in the concentration of its substrate phosphatidylinositol-4,5-biphosphate is rapidly detected in whole cell extract (Niebuhr et al., 2002), and a range of global to local effects of the enzymatic activity of IpgD has been reported in the literature (Puhar et al., 2013; Mellouk et al., 2014), complicating the portrait. When it comes to events downstream of entry, reports of effectors acting locally have been scarcer, although a few examples have emerged.

Recently, the fragmentation of the golgi apparatus of infected epithelial cells was reported independently by two research groups (Mounier et al., 2012; Burnaevskiy et al., 2013). A newly identified effector named IpaJ has been implicated in this phenotype (Burnaevskiy et al., 2013, 2015; Dobbs et al., 2015). IpaJ does so using its cysteine protease activity to cleave the N-miristoyl modification of ARF1 and ARF2 (Burnaevskiy et al., 2013). In a latter study, the same group revealed that IpaJ demiristoylated a large group of host proteins during *in vitro* experiments, but that it was highly specific to Golgi apparatus-associated ARF/ARL small GTPases when delivered inside host cells through the T3SA (Burnaevskiy et al., 2015). Specificity was also mediated in part by the capacity of IpaJ to recognize the GTP-bound form of golgi associated ARFs. Other factors impacting on the subcellular localization of IpaJ could be implicated in the selections of its substrates, as it also recognizes the GTP-bound form of the PM-associated ARF6 although the N-miristoyl of this latter protein is not cleaved *in vivo*. These data support the notion that the specificity of IpaJ enzymatic activity may come from its secretion site, where only a limited number of its potential N-miristoylated substrates are accessible, rather than from the specialization of its catalytic site to bind only a subset of N-miristoylated proteins. It is noteworthy though that the *in vivo* identification of IpaJ substrates was performed at 6 h post-entry (Burnaevskiy et al., 2015). It would be interesting to check if IpaJ is processing alternative N-miristoylated targets more early during the infection process when both its distribution and concentration should be very different, as one would hypothesize based on our model.

Autophagy is the process that leads to capture, classically in a double membrane compartment, and degradation of cytoplasmic content in response to specific metabolic cues. ATG8/MAPLC3 (LC3) proteins are canonical marker of autophagosomes. A subset of autophagy called xenoautophagy is used as a countermeasure against foreign particles such as viruses and bacteria (Baxt et al., 2013; Huang and Brumell, 2014). *S. flexneri* had been previously shown to resist xenoautophagy using its effectors IcsB and VirA (Ogawa et al., 2005; Dong et al., 2012). Harnessing the power of the TSAR system to distinguish *S. flexneri* intracellular sub-populations (Campbell-Valois et al., 2014b), we recently provided evidence that IcsB and VirA are both acting in the vicinity of actively secreting bacteria during cell-to-cell spread (Campbell-Valois et al., 2015). IcsB, VirA, and LC3 relocated around secreting bacteria in this context. A higher proportion of *icsB* and *virA* than wild type bacteria were LC3 positive during cell-to-cell spreading. *icsB virA* double mutant strain was even more attenuated than the single mutants in a plaques formation assay, displaying strongly diminished cell-to-cell spreading capacity. Moreover, *icsB virA* mutated bacteria were trapped in LAMP2 positive compartments from which they could hardly escape. These results suggested that IcsB and VirA are acting in synergy to allow escape from LC3 positive compartments formed during cell-to-cell. LC3 is also recruited around secreting bacteria during entry, but escape from the LC3 positive compartments in this context seems to be relatively independent of IcsB and VirA, although time of residence in the entry vacuole might be slightly extended for *icsB virA* bacteria (Campbell-Valois et al., 2015). Taking advantage of LC3 recruitment during entry, we demonstrated that LC3-positive entry vacuoles containing actively secreting bacteria were composed of a single membrane, as in the process of LC3-associated phagocytosis (LAP) previously reported in many bacterial pathogens (Lai and Devenish, 2012; Huang and Brumell, 2014). Therefore, we concluded that: (1) LC3 is recruited directly to existing bacteria-containing vacuoles; (2) vacuolar bacteria concomitantly secrete IcsB and VirA; (3) IcsB and VirA associate transiently with the vacuolar membranes, (4) but they act locally to favor escape from LC3-positive compartments most significantly during cell-to-cell spread (Figure 4B). Observations that wild type bacteria failed to complement in trans the deficiency of *icsB virA* bacteria support this model (Campbell-Valois et al., 2015). The action of IcsB and VirA in the vicinity of secreting bacteria is also supported by evidence that *icsB icsA, virA icsA*, and *icsB virA icsA* strains, which are all confined to the cytoplasm due to cell-to-cell spread deficiency, did not significantly recruit LC3 (Campbell-Valois et al., 2015). Although IcsB and VirA are acting in synergy, they have been shown to act on apparently unrelated targets (Ogawa et al., 2005; Dong et al., 2012). VirA is a Rab GTPase activating Protein (GAP) with Rab1-GTP being the most efficiently catalyzed substrate, although other Rabs (e.g., Rab33 and Rab35) are also processed efficiently (approximately three-fold less than Rab1) (Dong et al., 2012). The recruitment of Rab1 to bacteria containing vacuoles, phagosome, and autophagosome (Ingmundson et al., 2007; Zoppino et al., 2010; Huang et al., 2011; Campbell-Valois et al., 2012) suggests that Rab1 could be recruited as well to vacuoles containing *S. flexneri* rendering it available to neighboring VirA that would have been freshly delivered through T3SA (Campbell-Valois et al., 2015). IcsB has been suggested to protect *S. flexneri* from autophagy by shielding IcsA from direct recognition by ATG5, a component of the autophagy pathway (Ogawa et al., 2005). Other results rather suggest that the role of IcsA in LC3 recruitment is indirect through the formation of cell-to-cell spread vacuoles that, as phagosome-like compartments, could be subject to LAP (Campbell-Valois et al., 2015). This alternative model would readily explain LC3 recruitment during entry, but its absence at later stages of infection in *icsA* strains (Baxt and Goldberg, 2014; Campbell-Valois et al., 2015). A cholesterol-binding domain was also identified in IcsB and showed to be essential for the ability of IcsB to enable autophagy escape (Kayath et al., 2010). Cholesterol being putatively found in abundance in the Golgi apparatus, the PM and in compartments such as early phagosomes derived from it (van Meer et al., 2008), freshly secreted IcsB could act directly using its cholesterol binding domain on the membrane of *S. flexneri*-containing vacuole. Interestingly, interruption of cholesterol flux inside macrophages have been shown to block fusion of phagosomes with lysosomes (Huynh et al., 2008). As yet, there are still many unknowns concerning the targets and modes of action of IcsB and VirA that enable escape from LC3-positive vacuoles. In particular, how IcsB and VirA activities synergize in that context is completely unknown.

An interesting example of how intrinsic properties of a given effector could impact on its range of action was recently reported for OspG. OspG is endowed with Ser/Thr kinase activity; it binds specifically to E2 ubiquitin conjugating enzyme (e.g., UbCH5, UbCH7) loaded with ubiquitin (E2~Ub) and blocks IκBα degradation induced by tumor necrosis factor-α (TNFα, Kim et al., 2005). It has also been shown to bind free ubiquitin and polyubiquitin chains (Zhou et al., 2013), although E2~Ub seems to bind OspG with more affinity and increases its kinase activity more readily than free ubiquitin (Grishin et al., 2014; Pruneda et al., 2014). OspG is an atypical Ser/Thr kinase with a shorter primary structure than its eukaryotic counterparts (Kim et al., 2005; Grishin et al., 2014; Pruneda et al., 2014). Structures of E2~Ub-OspG complexes were recently reported (Grishin et al., 2014; Pruneda et al., 2014). OspG is binding at the intersection of the ubiquitin C-terminus and the catalytic site of the E2 to which the latter is tethered, hence contacting both proteins constituting the E2~Ub complex. OspG adopts the active conformation of Ser/Thr kinases (Grishin et al., 2014; Pruneda et al., 2014). Both studies showed that disrupting interfaces between OspG and E2~Ub abrogated the capacity of OspG to decrease IkBα degradation (Grishin et al., 2014; Pruneda et al., 2014). What is particularly interesting for the main matter discussed here, is that mutants in the primary structure of OspG disrupting its capacity to interact with E2~Ub have a much shorter half-life than the wild type within host cells (Grishin et al., 2014). This observation suggests that integration of OspG in a ternary complex with E2~Ub stabilizes its structure and/or protect it from proteases. The range of action of OspG upon secretion is therefore likely regulated by its binding affinity to E2~Ub and the fraction of OspG found in the complex with E2~Ub at any given time. Assuming that E2~Ub concentration is relatively homogenous across the cytoplasm, one can assume that the likelihood of forming the tripartite complex will be maximal in the vicinity of secreting bacteria where OspG concentration would be higher. As OspG, either free or in the tripartite complex, diffuses away, its effective concentration will decrease thereby mechanically reducing the fraction found in the stabilizing tripartite complex. In consequence, the concentration of free OspG will be higher further down its diffusion gradient leading to reduced activity and degradation. Therefore, this phenomenon will effectively restrain OspG capacity to act at long distances. Nevertheless, many aspects of the interplay between OspG, its kinase activity, the E2~Ub complex and the degradation of IκBα remain to be understood.

### Counterexamples: effectors acting at a long distance from their secretion site

*Shigella* spp. possess 12 *ipaH* genes, but due to pseudogenes and gene duplications they give rise to a maximum of 9 distinct proteins across *Shigella* spp. (Bongrand et al., 2012). IpaHs are E3 ubiquitin ligases (Rohde et al., 2007; Singer et al., 2008; Zhu et al., 2008), and the search for their host targets has attracted considerable interest (Rohde et al., 2007; Ashida et al., 2010, 2013, 2014; Wang et al., 2013; Suzuki et al., 2014; Tanner et al., 2015). The substrates identified so far are molecules implicated in inflammatory pathways converging on NFκb. Most of these validated targets (e.g., NEMO, NFκB p65 etc.) are cytoplasmic proteins that have not been reported in these studies to physically associate or to relocate to *S. flexneri*-containing protrusions or vacuoles where secretion is actively taking place (Campbell-Valois et al., 2014b). The single exception might be glomulin, which is degraded by the proteasome in an IpaH7.8-dependent manner in macrophages (Suzuki et al., 2014). In this study, glomulin was found in the vicinity of *ipaH7.8* bacteria only. It is not clear though if IpaH7.8 is ubiquitylating glomulin specifically around secreting bacteria or away of bacteria, hence preventing its recruitment to cytoplasmic bacteria. Since there have not been many studies on glomulin reported in the literature, further work will help shedding light on its role during bacterial infection.

OspF is arguably the prototypical example of effectors acting at a long distance from their secretion site. Indeed, OspF is a phosphothreonine lyase that specifically removes the O-phosphate group from the threonine of the activation loop of MAP kinases (i.e., Thr183 in Erk1) (Li et al., 2007). This modification irreversibly inactivates the MAPK (e.g., ERK1/2, p38 etc.), blocks the activation of the interleukin-8 promoter by the NFκb pathway and strongly dampens the inflammatory response (Arbibe et al., 2007). These events are taking place in the nucleus, where most phosphorylated ERK1/2 are found. OspF also spontaneously locates to the nucleus upon transfection of tissue culture cells (Arbibe et al., 2007) (Figure 4B). OspF is sufficiently small (~28 kDA) to freely diffuse in the nucleus and it does not display a typical basic nuclear localization sequence within its primary structure. OspF could be anchored to the appropriate sites in the nucleus through binding to Heterochromatin Protein 1 γ (HP1γ) (Harouz et al., 2014), which is historically associated with heterochromatin formation but has also been associated with transcriptionally active loci such as the IL-8 promoter. Supporting the important role of OspF in the nucleus, the *ospF* mutant modulates the transcription of more genes than other mutant strains tested including *mxiE* strain, which lacks expression of second-wave effectors (Parsot, 2009). In addition, affected genes are attributed to three distinct pathways: inflammation, apoptosis and stress response, going way beyond its classical role in dampening the inflammatory response (Lippmann et al., 2015). OspB is another effector that is located to the nucleus and that could be implicated in modulating the inflammatory response, potentially coordinating its action with OspF (Zurawski et al., 2009; Ambrosi et al., 2015). Nevertheless, even in the case of effectors shown to be acting at a long distance, it is impossible to discard the possibility that they have also a local function that has not been uncovered yet (Figure 4B).

## Are T3SA in other bacteria also spatio-temporally regulated?

**Figure 5:**
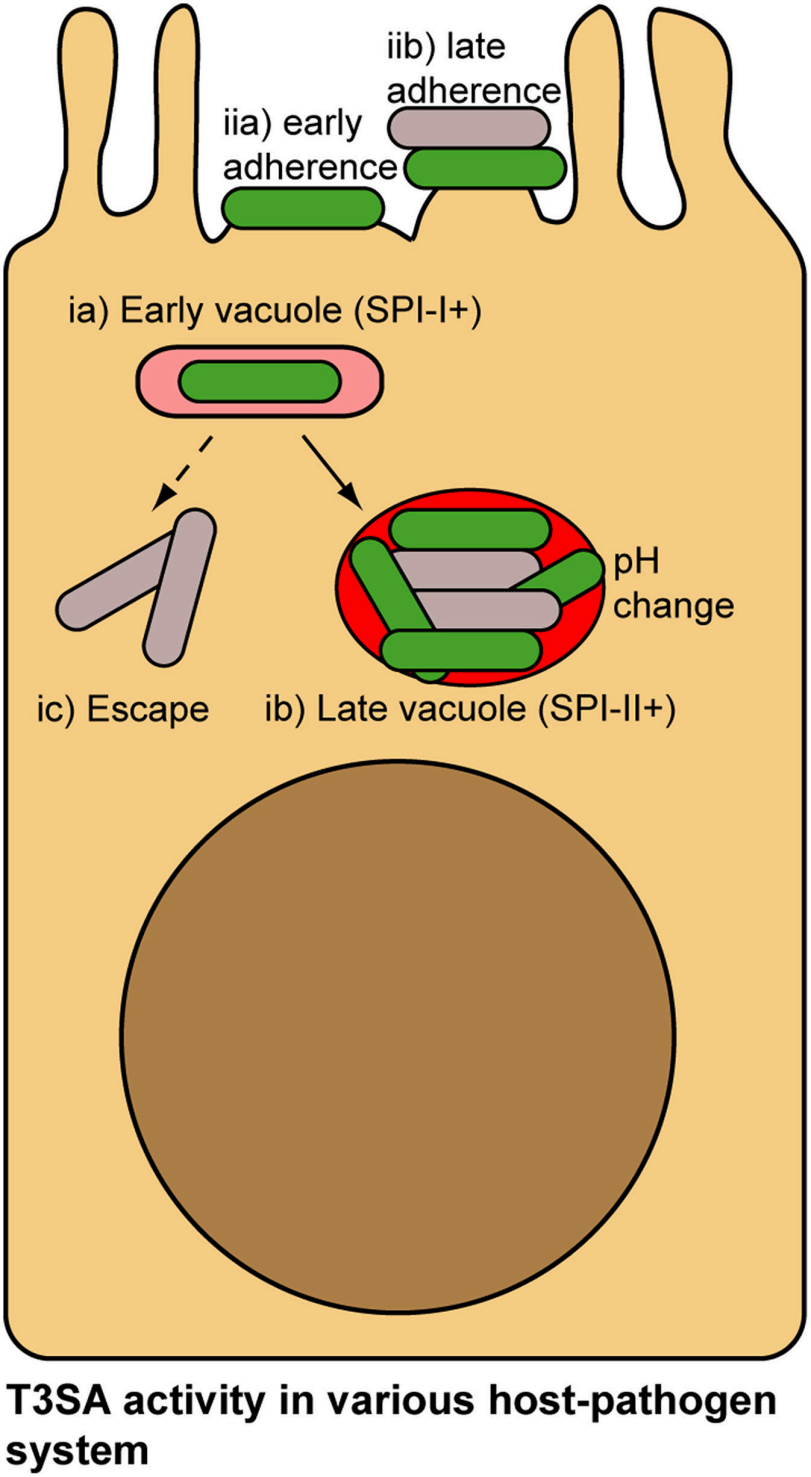
**Alternative lifestyles of pathogenic bacteria associated with host cells and potential mechanisms of regulation of their T3SA**. *Salmonella* invade epithelial cells using Salmonella Pathogenicity Island-1 (SPI-1) T3SA **(ia)**. pH change in the vacuole and concomitant sensing of cytosolic pH induce activation of Salmonella Pathogenicity Island-2 (SPI-2) T3SA in *Salmonella* **(ib)**. As bacteria accumulate in vacuole, they are not in contact with the membrane, which would probably inactivate SPI-1 and SPI-2 T3SA **(ib)**. Occasionally Wt *Salmonella* escape their vacuole and access the cytoplasm. Loss of contact with vacuolar membrane in this case could also potentially lead to T3SA inactivation **(ic)**. EPEC adhesion to the PM of epithelial cells proceeds in multiple stages (e.g., bundling forming pili-, T3SS/Tir- and EspA-dependent, etc.), culminating in the formation of actin-rich pedestals structure at bacterial adhesion point. Throughout this adhesion process, activity of the T3SA could be modulated **(iia,b)**. In addition, within microcolonies some bacteria will occasionally loose contact with the PM, which similarly to the previous example could inactivate T3SA. Secreting bacteria, green; non-secreting bacteria, gray.

Due to the “invade and evade” infectious strategy used by *S. flexneri* (i.e., successive PM- and cytoplasm association), spatio-temporal regulation of its T3SA is a plausible mode of action. Since *Burkholderia mallei* and *Burkhloderia pseudomallei* have also adopted a similar infectious strategy (Stevens et al., 2006; Gong et al., 2011), their T3SA is probably regulated similarly to *S. flexneri*. What about other type of T3SA-expressing pathogens? Two main alternative infectious strategies exist: (i) bacteria residing in vacuole, such as is the case with *Salmonella* spp. or *Chlamydia* spp.; (ii) bacteria associating with the extracellular face of the PM in a transient (*Yersinia* spp.) or stable fashion (enteropathogenic *E. coli* and *Citrobacter rodentium*) (Figure 5). Although they are considered paradigmatic vacuolar pathogens, *Salmonella* spp. are not only found in large vacuoles and tighter tubular compartments, but also in the cytoplasm (LaRock et al., 2015; Liss and Hensel, 2015). These bacteria use T3SA encoded by the Salmonella Pathogenicity Island-1 (SPI-1) to invade epithelial cells. It was shown that acidification of the bacteria-containing vacuole and ensuing sensing of neutral pH of the cytoplasm through its translocon led successively to Salmonella Pathogenicity Island-2 (SPI-2) T3SA assembly and activation (Yu et al., 2010), which is important for shaping the vacuolar niche of this pathogen (LaRock et al., 2015; Liss and Hensel, 2015). Nevertheless, whether bacteria that are located in the middle of large vacuoles and in which the T3SA is not directly contacting the host membrane are actively secreting or not is currently unknown. As infection progresses, the evolution of these vacuoles into tight tubular compartments (LaRock et al., 2015; Liss and Hensel, 2015) might allow membrane-bound *Salmonella* to maintain lasting SPI-2 T3SA activities. Another possible opportunity for inactivation of SPI-1 and SPI-2 T3SA could happen in cytoplasmic bacteria, which represent between 6 and 51% of intracellular bacteria depending on the stage of infection (Knodler et al., 2014). Enteropathogenic *E. coli* (EPEC) and *C. rodentium* associate stably with the PM through the formation of pedestals structure by secreting their own receptor Tir (Kenny et al., 1997; Mundy et al., 2005). As yet, methods developed to measure secretion activity have not shown regulation of T3SA activity following initial activation (Charpentier and Oswald, 2004; Mills et al., 2008, 2013; Yerushalmi et al., 2014). The different stages of adhesion in EPEC (e.g., bundling forming pili-, T3SS/Tir-, and EspA-dependent), leading progressively to more intimate interactions between bacteria and the host PM might nonetheless represent circumstances where the T3SA activity would be modulated (Cleary et al., 2004). Studies about T3SA regulation mechanisms in these bacterial pathogens and others would certainly benefit from the development of secretion activity reporters as well (Campbell-Valois and Sansonetti, 2014).

## Conclusions

Many studies discussed above addressed how the regulated secretion of bacterial effectors impacts on their subcellular distribution, concentration, and function. Such observations could have important consequences. For example, part of effectors specificity could stem from their location rather than from the evolution of their catalytic site to accommodate a more restrained group of substrates. Historically, experimental approaches employed to determine host protein targets of bacterial effectors have been relying mostly on yeast-two-hybrid screens and overexpression in tissue culture cells. Although the legacy of these approaches in host-pathogen interactions is considerable, they are not optimal to find host targets that are selected on the basis of their location at or around actively secreting bacteria. Novel experimental strategies will have to be developed to tackle these questions.

## Author contributions

FXCV wrote the initial and final version. SP contributed ideas and wrote the final version.

### Conflict of interest statement

The authors declare that the research was conducted in the absence of any commercial or financial relationships that could be construed as a potential conflict of interest.
